# Supplementary figures and images for: Non-trophic interactions strengthen the diversity—functioning relationship in an ecological bioenergetic network model
Source: PLoS Comput Biol. 2019 Aug 29;15(8):e1007269. doi: 10.1371/journal.pcbi.1007269 (PMC6715155; doi:10.1371/journal.pcbi.1007269)

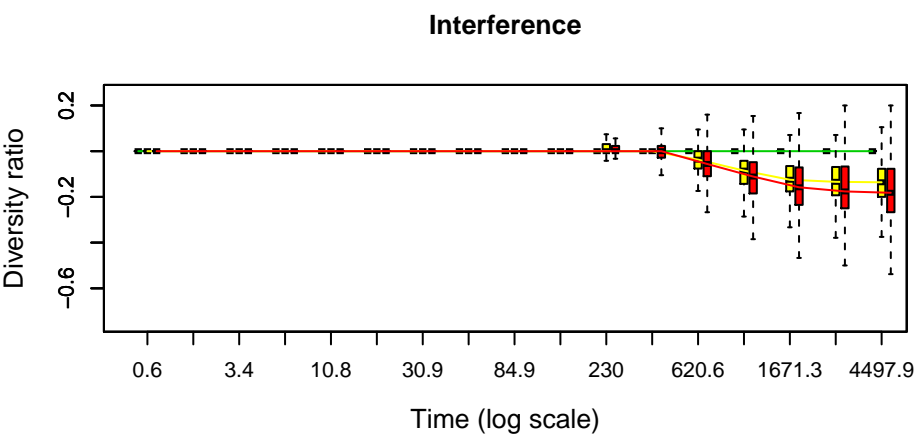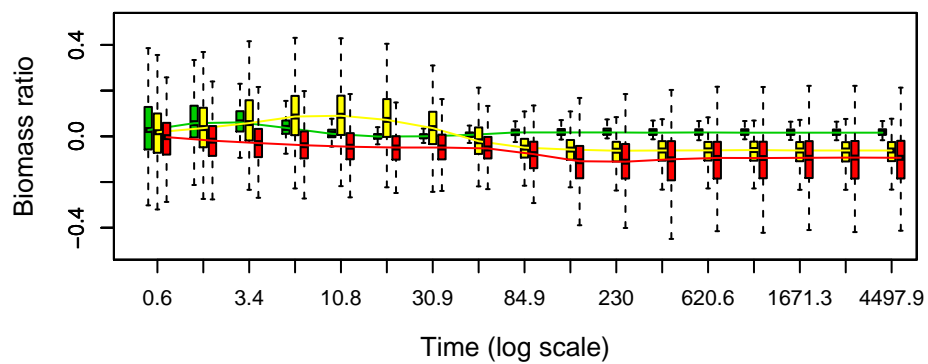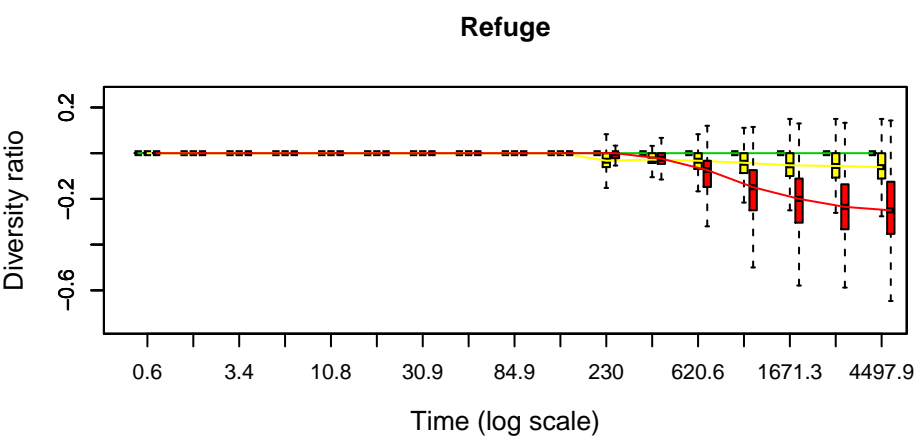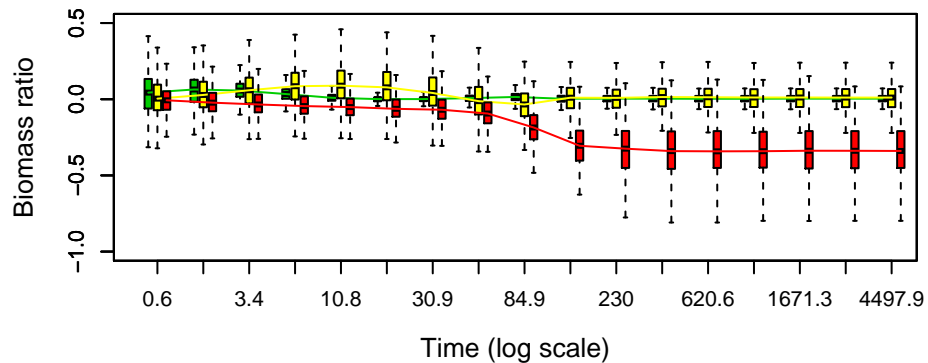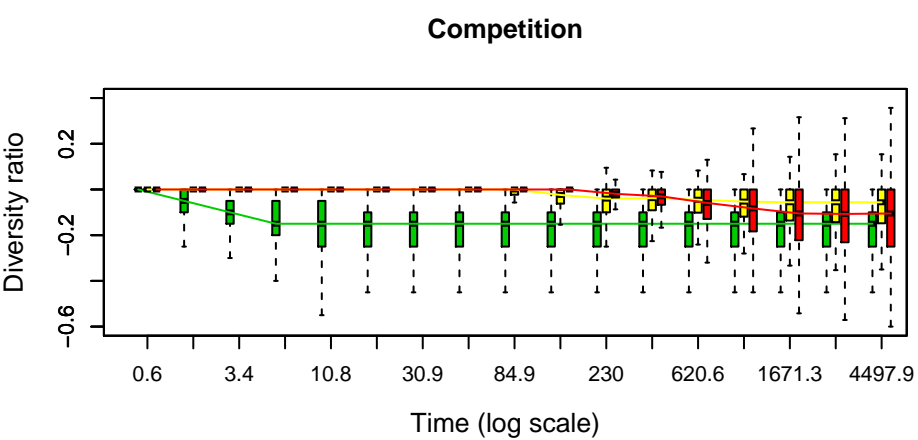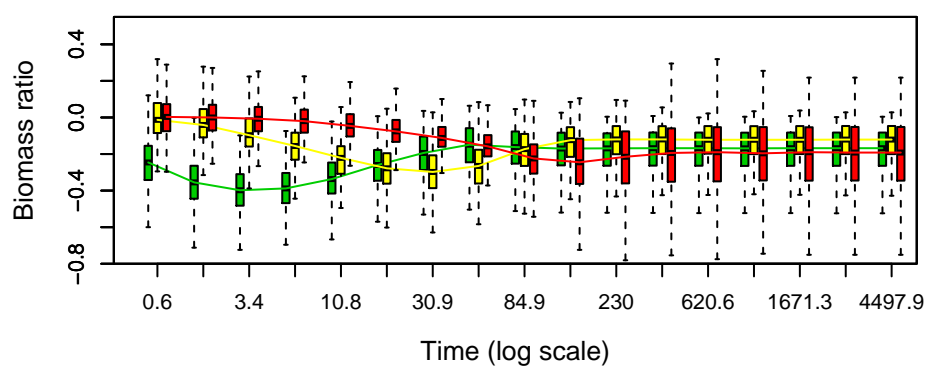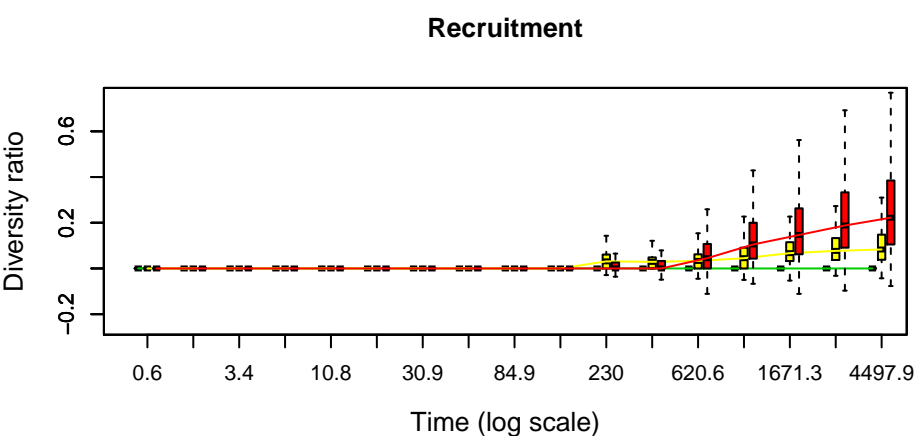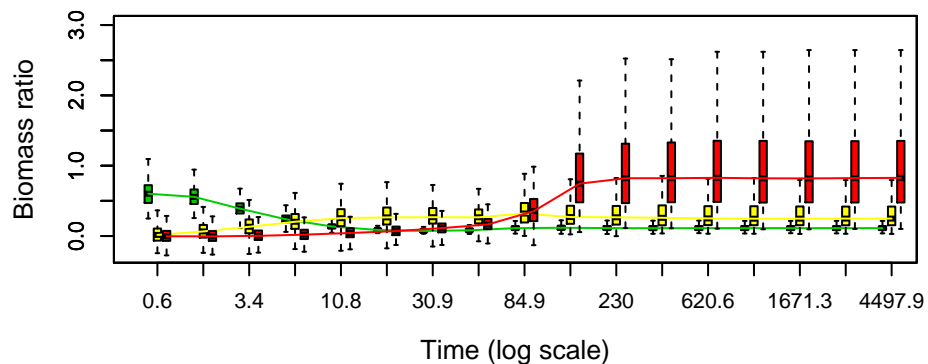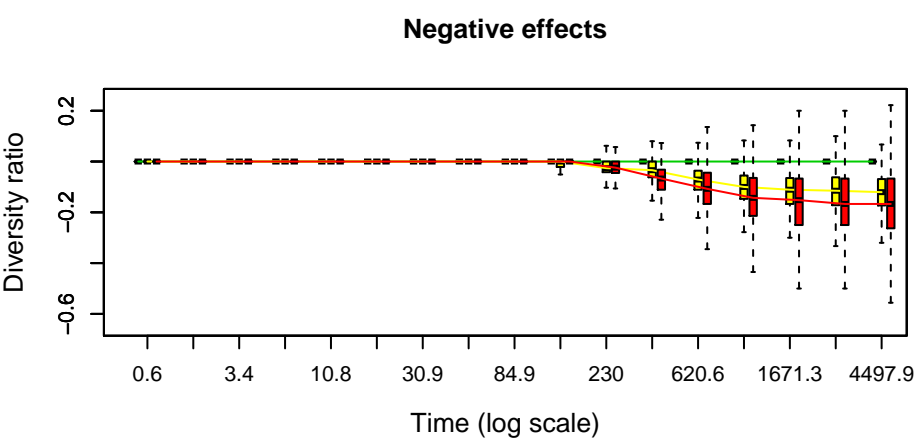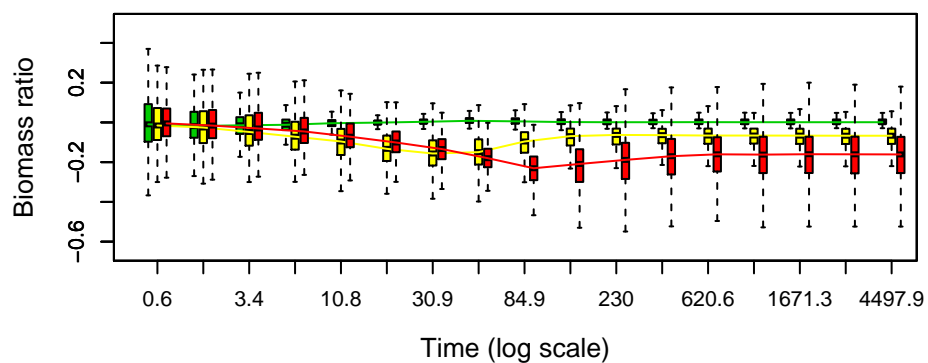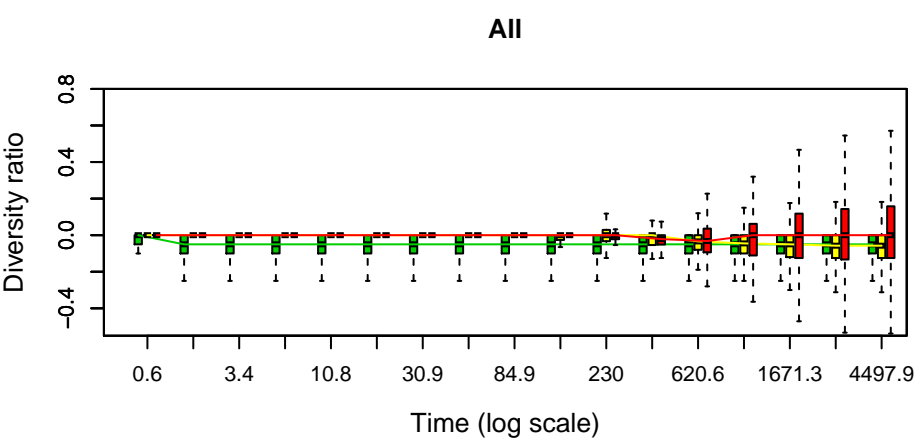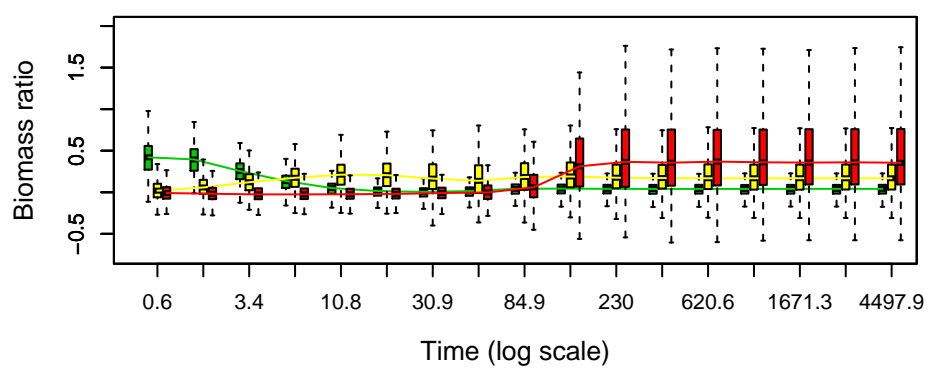

Supplement: S1 Fig — Each simulation starts with 600 trophic links and 100 non-trophic links. Note that on the x-axis, time is on a log-scale. Green: TL = 1, yellow: TL between 2 and 3, red: TL> 3. TL refers to the prey-averaged trophic level measured as one plus the mean trophic level of all the species resources, where the trophic level of a resource is the chain length from the resource to a basal species [59]. Species whose TL is 1 are primary producers. (PDF) [file pcbi.1007269.s001.pdf]

# Diversity

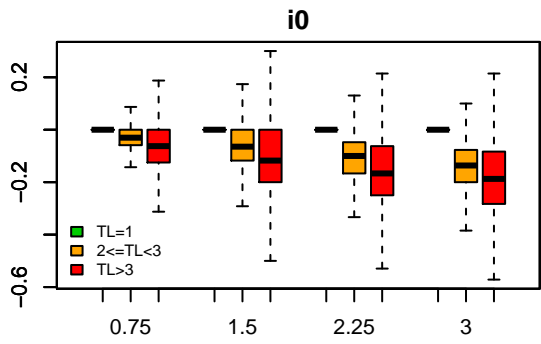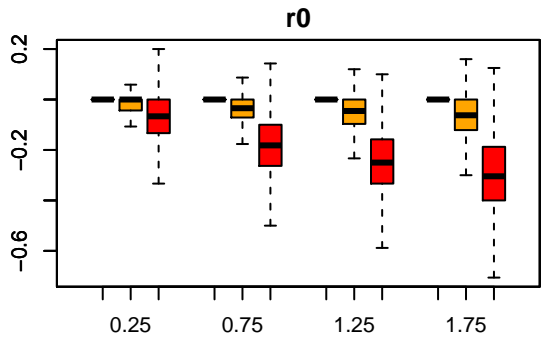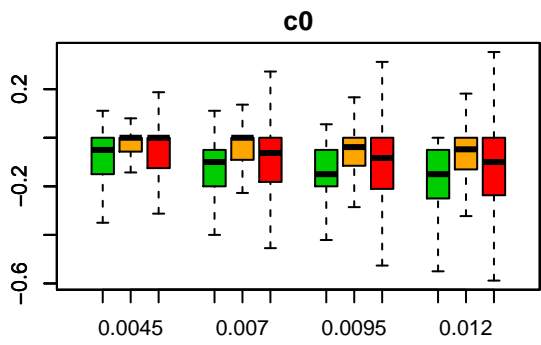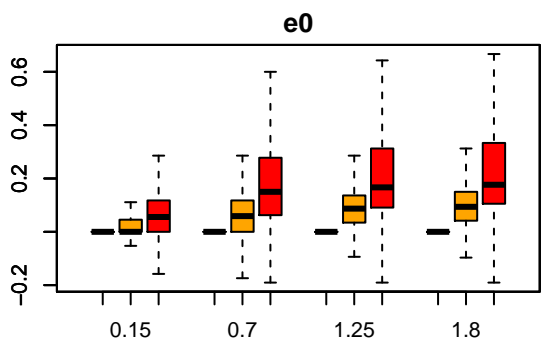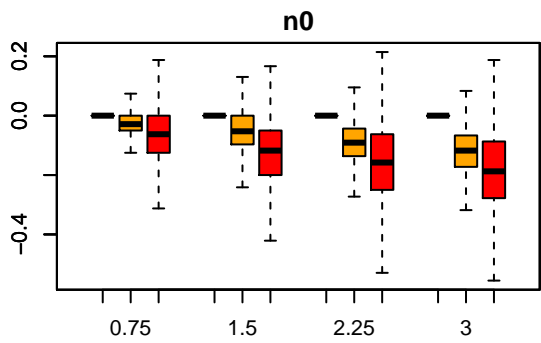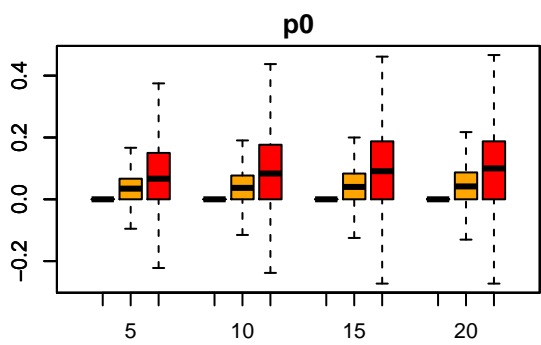

# Biomass

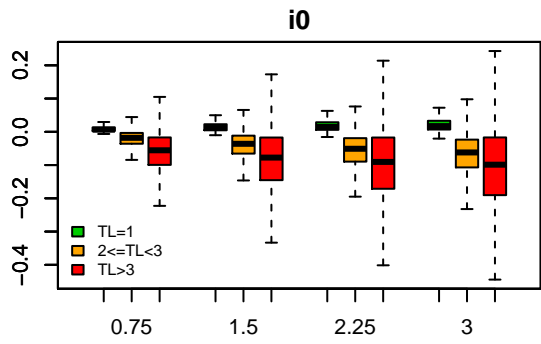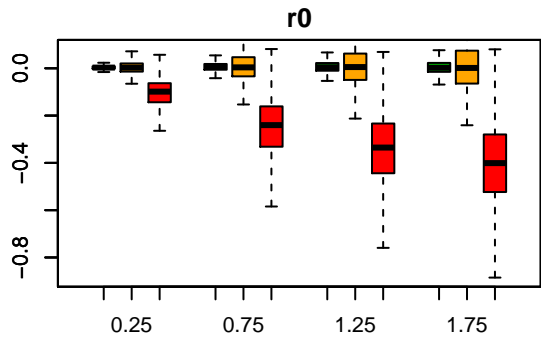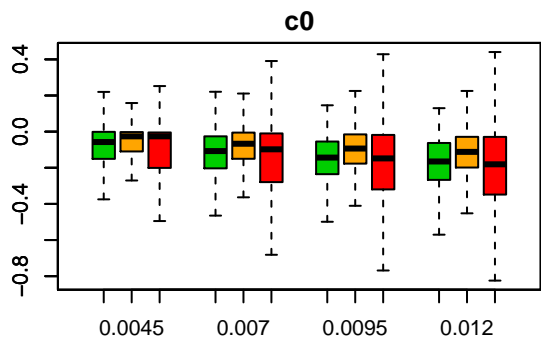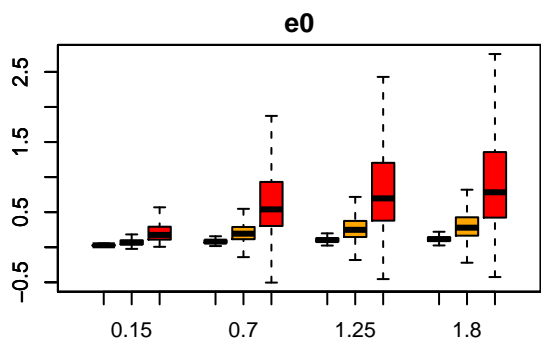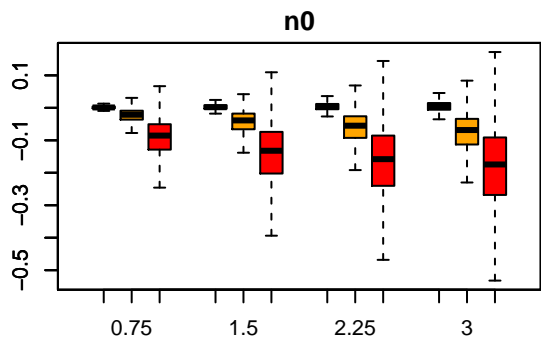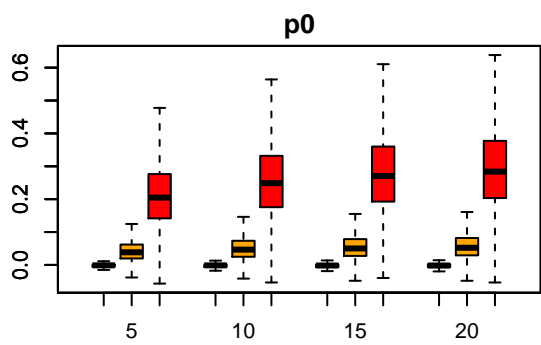

# Production

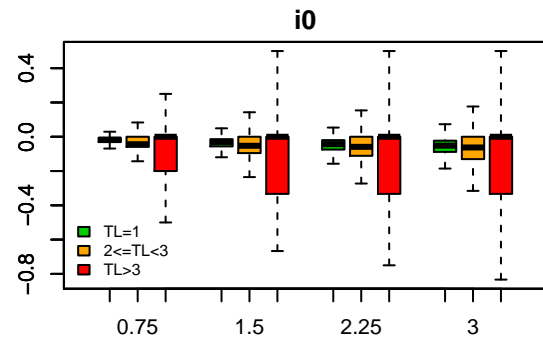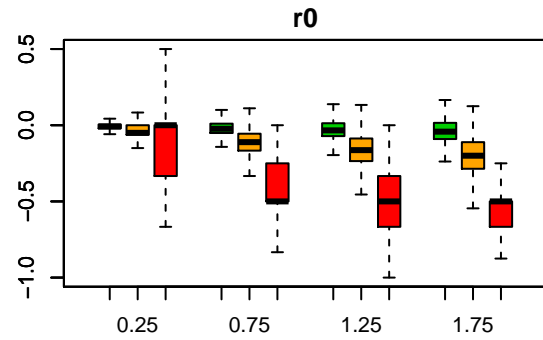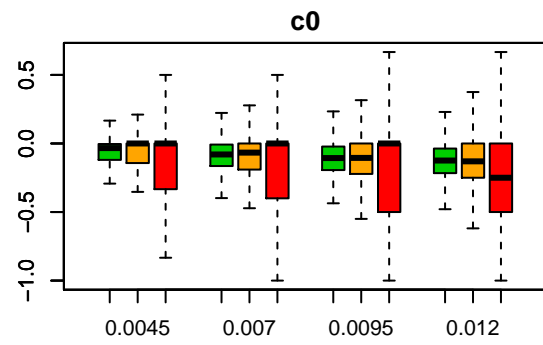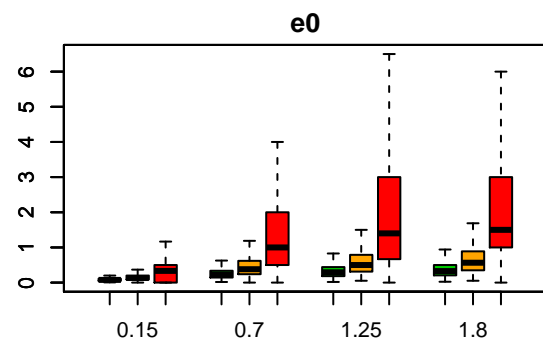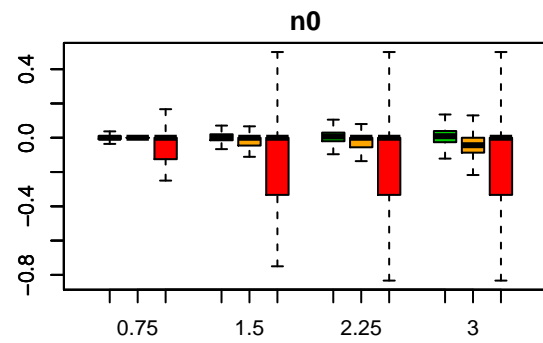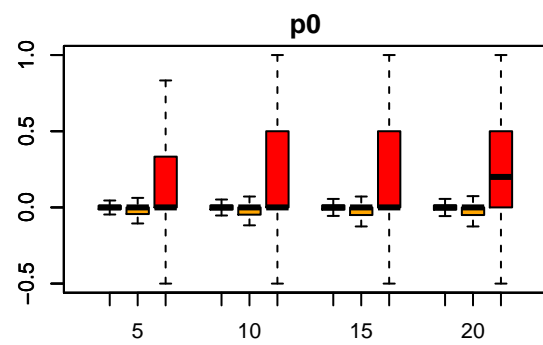

Supplement: S2 Fig — Note that each NTI has its own y-axis. Green: TL = 1, yellow: TL between 2 and 3, red: TL> 3. TL refers to the prey-averaged trophic level measured as one plus the mean trophic level of all the species resources, where the trophic level of a resource is the chain length from the resource to a basal species [59]. Species whose TL is 1 are primary producers. Note that the y-axis differ for the different NTIs. (PDF) [file pcbi.1007269.s002.pdf]

**1 I + 1/2 E**

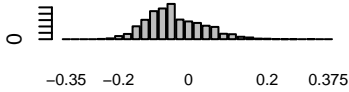

**1 I + 1 E**

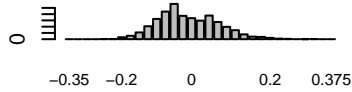

**1/2 I + 1 E**

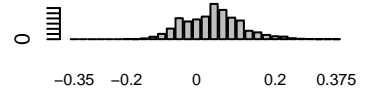

**1 N + 1/2 E**

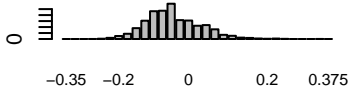

1 N + 1 E

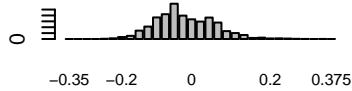

**1/2 N + 1 E**

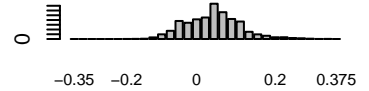

**1 R + 1/2 E**

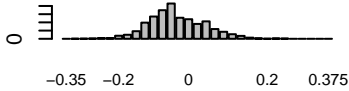

**1 R + 1 E**

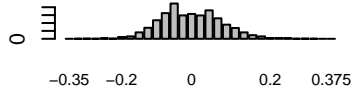

**1/2 R + 1 E**

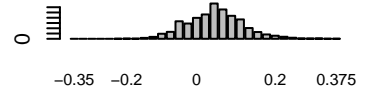

**1 C + 1/2 E**

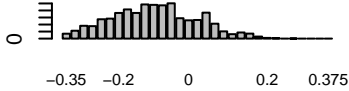

**1 C + 1 E**

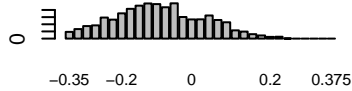
$$1/2 \text{ C} + 1 \text{ E}$$
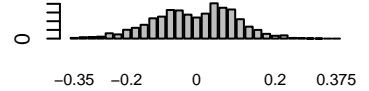
$$1 (I+N+R+C) + 1/2 E$$
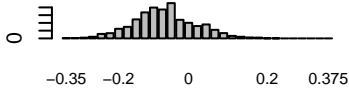
$$1 (I+N+R+C) + 1 E$$
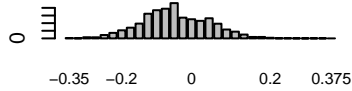
$$1/2 (I+N+R+C) + 1 E$$
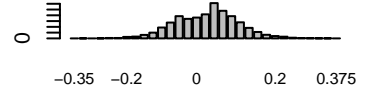

Supplement: S3 Fig — The intensity of each NTI type is now fixed and we vary the relative number of links of different NTI types when put together. The left column correspond to situations where there are twice more detrimental than beneficial links, the middle column shows results with equal number of beneficial and detrimental links, and the right column correspond to cases where there are twice more beneficial than detrimental links. The panels correspond to different combinations of NTIs: I for interference, E for recruitment facilitation, N for negative effects on mortality, R for refuge, C for competition. At fixed intensity and with equal number of links, detrimental links tend to take over (slightly). There are configurations of relative abundance of the different types of NTIs in which positive and negative effects can balance each other. (PDF) [file pcbi.1007269.s003.pdf]

**slope= 0.027**

Production with NTI

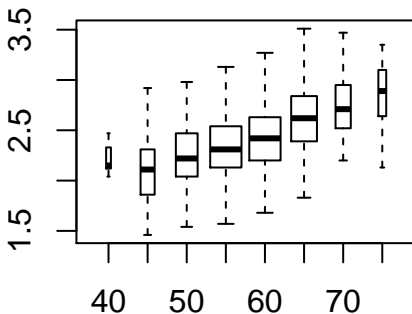

Diversity with NTI

Supplement: S4 Fig — Same as Fig 3B but for production instead of biomass. The slope obtained by linear regression of the relationship is indicated on top of the panel. (PDF) [file pcbi.1007269.s004.pdf]
