# Supplementary material for: Non-trophic interactions strengthen the diversity—functioning relationship in an ecological bioenergetic network model
Source: PLoS Comput Biol. 2019 Aug 29;15(8):e1007269. doi: 10.1371/journal.pcbi.1007269 (PMC6715155; doi:10.1371/journal.pcbi.1007269)
Supplement: S5 Fig — See Methods, part ‘The dynamical model’, for more details about the parameters and how they contribute to the dynamical equations. Independently of the combinations of parameter values found, the slope of the BEF is stronger in the presence than in the absence of NTIs. (PDF) [file pcbi.1007269.s005.pdf]

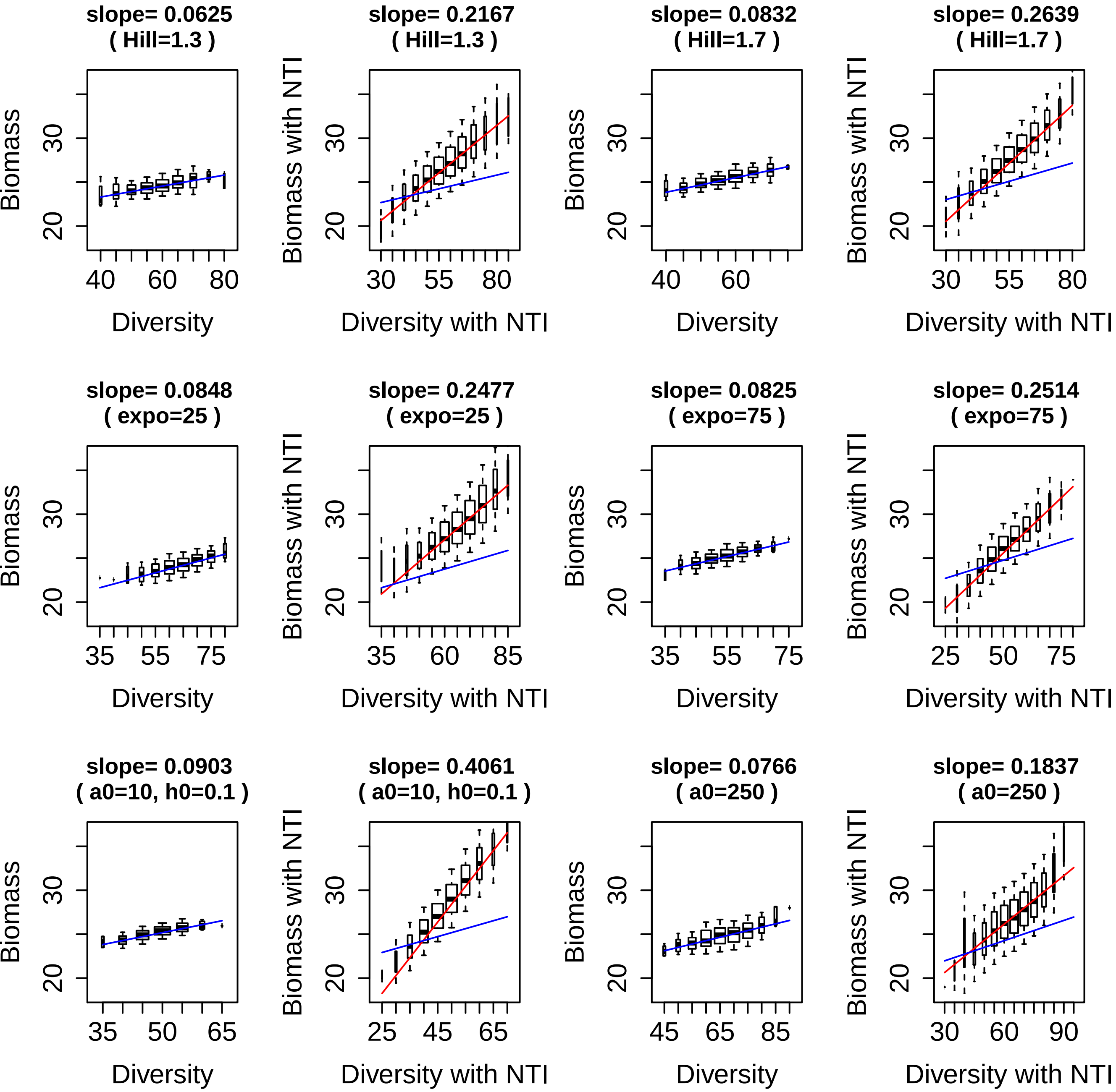
slope= 0.0625
(Hill=1.3)
Biomass
Diversity
slope= 0.2167
(Hill=1.3)
Biomass with NTI
Diversity with NTI
slope= 0.0832
(Hill=1.7)
Biomass
Diversity
slope= 0.2639
(Hill=1.7)
Biomass with NTI
Diversity with NTI
slope= 0.0848
(expo=25)
Biomass
Diversity
slope= 0.2477
(expo=25)
Biomass with NTI
Diversity with NTI
slope= 0.0825
(expo=75)
Biomass
Diversity
slope= 0.2514
(expo=75)
Biomass with NTI
Diversity with NTI
slope= 0.0903
(a0=10, h0=0.1)
Biomass
Diversity
slope= 0.4061
(a0=10, h0=0.1)
Biomass with NTI
Diversity with NTI
slope= 0.0766
(a0=250)
Biomass
Diversity
slope= 0.1837
(a0=250)
Biomass with NTI
Diversity with NTI
